# Access to High-Cost Biological Agents: Perceptions of Brazilian Patients with Inflammatory Bowel Diseases

**DOI:** 10.3390/jcm12072672

**Published:** 2023-04-03

**Authors:** Rogerio Serafim Parra, Sandro da Costa Ferreira, Vanessa Foresto Machado, Cintia Maura Caseiro Nigro, José Joaquim Ribeiro da Rocha, Luiz Ernesto de Almeida Troncon, Omar Feres

**Affiliations:** 1 Department of Surgery and Anatomy, Ribeirão Preto Medical School, University of São Paulo, Ribeirão Preto 14048-900, Brazil; 2 Department of Clinical Medicine, Ribeirão Preto Medical School, University of São Paulo, Ribeirão Preto 14048-900, Brazil

**Keywords:** inflammatory bowel diseases, ulcerative colitis, Crohn’s disease, patient-reported outcomes, biological therapy

## Abstract

Background and aims: Brazilian patients with inflammatory bowel diseases (IBD) requiring therapy with biological agents usually have access to medicines through the National Unified Health Care System (SUS). This study aimed to analyze Brazilian IBD patient perception regarding access (availability and provision quality) to high-cost drugs in the public health care system. Methods: A questionnaire-based survey was carried out in an IBD referral center in Brazil. All adult patients with an established diagnosis of ulcerative colitis (UC) or Crohn’s disease (CD) that use biological therapy were invited to participate. Data were collected on the biological in use, lack of distribution (number of absences, average time to regularization, impairment in patient treatment), and difficulties reported by patients in obtaining the drugs. Results: Overall, 205 patients met the inclusion criteria and answered the questionnaire. Most of the patients had CD (*n* = 161, 78.5%), nearly half of them (*n* = 104, 50.7%) were female; 87 patients (42.4%) were unemployed, and of these, 40 patients (19.5%) had government assistance as the main source of income. Regarding the medications used, infliximab (*n* = 128, 62.5%) was the most used medication, followed by adalimumab (*n* = 39, 19.0%). Most patients (*n* = 172, 83.9%) reported at least one failed delivery of biological medicine in the last year, with a single shortage in forty-two patients (24.4%), at least two shortages in forty-seven patients (27.3%), and three or more shortages in seventy-eight patients (45.3%). The average time to regularize the distribution was up to 1 month in 44 cases (25.6%), up to 2 months in 64 cases (37.2%), and more than 3 months in 56 patients (32.6%). Among patients who reported delays, 101 patients (58.7%) felt that it may have impaired their treatment. Conclusion: Brazilian IBD patients reported high rates of failure to dispense biological drugs by the national healthcare system within one year. Our data highlight the need for improvement in this system for the correct supply of medication to avoid treatment failure and relapse.

## 1. Introduction

Inflammatory bowel disease (IBD), such as Crohn’s disease (CD) and ulcerative colitis (UC), are chronic inflammatory conditions of the gastrointestinal tract, and their incidence and prevalence are rising across developing countries, including Brazil [1,2]. Both diseases are characterized by relapsing–remitting symptoms, have adverse impacts on patient quality of life, and are associated with a high-cost burden, especially in patients with moderate to severe disease [3,4,5,6]. Patients with CD can develop complications such as stenosis, fistulas, and abscesses, and the risk of surgical resection within ten years of diagnosis is up to 50%. In UC, the long-term complications include dysplasia, colorectal cancer, and stenosis, and patients with moderate to severe disease may be at risk of hospitalization and surgery [7,8,9].

The management of moderate to severe IBD has undergone significant changes over the past two decades due to the regulatory approval of the first biological drugs, i.e., infliximab [10]. Recently, several biological drugs, including other anti-tumor necrosis factor (anti-TNF) drugs (adalimumab, certolizumab pegol, and golimumab), infliximab and adalimumab biosimilars, anti-integrin (vedolizumab) and anti-interleukin (ustekinumab and risankizumab), and small molecules, such as tofacitinib and upadacitinib, have been approved, thus enriching the therapeutic arsenal for both diseases [11,12]. Medical management aims to achieve and maintain long-term remission and heal the gut mucosa, thus reducing morbidity and mortality and delaying the progression of the disease and associated complications, such as hospitalizations, work disability, and the need for surgery or ostomies [13]. Current treatment guidelines recommend early intervention with immunomodulators and/or biologics in high-risk patients with a severe disease phenotype at presentation [14].

Currently, despite economic difficulties in Brazil, biological therapy for patients with IBD is made available by the National Unified Health Care System (SUS) through the Specialized Component of Pharmaceutical Assistance (CEAF), or through a request from health operators for those who have access to health insurance. Within the scope of the public system, the Ministry of Health carries out centralized purchases and forwards them to the states that carry out operational management [15]. Even though Brazil has three different classes of approved biological drugs (anti-TNF, anti-integrin, and anti-interleukin) and a JAK inhibitor (tofacitinib), access to biological/small molecule treatment is difficult, and there are differences between patients in the public and private health systems. A recent Brazilian study showed that there is difficulty in accessing or releasing medicines, particularly those associated with biological therapy. For CD, 70.4% of physicians responded that they had difficulty accessing or releasing biological medicines, and 72.3% responded that this was also the case with the other drug classes used in the treatment of the disease. The numbers for this variable regarding biological therapy for UC were higher (95.1%), and 57.7% of physicians answered that they had difficulties with other drugs [15]. There have been increasing legal actions against the government regarding the access and release of these medicines. In addition, there are many recent reports of a lack in the dispensing of biologicals in Brazil by the SUS. Therefore, the purpose of this study was to evaluate patient perception of the availability and access to high-cost drugs in the public health care system in an IBD referral center.

## 2. Materials and Methods

### 2.1. Study Design and Population

A questionnaire-based survey with 14 objective questions related to access to high-cost biologics was carried out between February 2022 and March 2022. All adult patients with an established diagnosis of UC or CD and using biological therapy with follow-up in an IBD referral center in Southeast Brazil (Clinical Hospital, Ribeirão Preto Medical School, University of São Paulo (HCFMRP-USP)) were invited to participate and answer in semi-structured interviews. In addition, demographic and clinical data, such as sex, age, work status, diagnosis (UC or CD), and the biological drug in use, were obtained. The inclusion criteria were adults (>18 years old), with a diagnosis of IBD (UC or CD), with a history of using biological therapy, or who had been trying to obtain biological dispensation for at least six months and with access to biological drugs through the SUS. The exclusion criteria were mental incapacity, unwillingness to participate, or language barriers precluding adequate understanding or cooperation with the interviewer. Patients in exclusive conventional therapy (5-ASA derivates, steroids, immunomodulators), which means those who were not using any biologics, were also excluded, except for patients with a prescription for a biological in the last six months.

#### Data Variables Obtained from the Questionnaire-Based Survey

During routine outpatient consultation, patients received a questionnaire-based survey with 14 objective questions related to access to high-cost biologics. The 14 questions included the following:

1. What was the biologic prescribed?
2. Is there a lack or delay in the initiation of continuing biologics?
3. If yes, what is the number of absences in delivering the biologics?
4. What is the average time to regularize the distribution of the biologics?
5. Do you fear that missing the biological infusion can cause impairment in your treatment or lead to complications?
6. Did the patient think that obtaining the biologics is a bureaucratic process?
7. Did the attendant or the pharmacist of the high-cost pharmacy solve doubts about the storage of the biologics and the next steps after obtaining the drug?
8. In the case of lack or delay in the distribution of the biologics, was there an explanation of the reason for the lack of the drug?
9. Was there an attempt to contact the high-cost pharmacy (by phone, message, or e-mail)?
10. If yes, was this contact not possible, or was it possible and easy, or possible and difficult?
11. Would it be easier to obtain biologics at the tertiary hospital after your medical appointment or at the infusion clinic immediately before the infusion?
12. Would it be easier to get the drug at the high-cost pharmacy and leave it for infusion at the tertiary hospital or the outside infusion clinic?
13. Do you know about the sponsored assistance program by the distributing pharmaceutical company of the medicine drug?
14. Do you believe that it is important to have the treatment initiation program by the laboratory producing the biologics?

All patients were invited to report comments at the end of the self-administered questionnaire.

### 2.2. Ethical Considerations

The current study was approved by the Hospital’s Ethics Committee of Ribeirão Preto Medical School, University of São Paulo (CAAE 05859218.3.0000.5440, #3.117.993), and this is part of the project evaluating patients with IBD (CD or UC) in follow-up at the HCFMRP-USP. All participants provided written informed consent before this study. In addition, the patients included in this study consented to a review of their medical records for research purposes by providing informed written consent to this publication and any other related publications. All procedures were conducted in accordance with the 1964 Declaration of Helsinki and its later amendments or comparable ethical standards.

### 2.3. Statistical Analysis

A descriptive statistical analysis was conducted using frequency, percentage, mean, and range to describe the variables. Fisher’s exact test was used to evaluate the nominal variables. Overall, 197 patients were expected to participate. This sample size allowed estimates of the representative population with a 95% confidence interval (CI) and a margin of error of <5%.

## 3. Results

### 3.1. Baseline Characteristics

**Figure 1:**
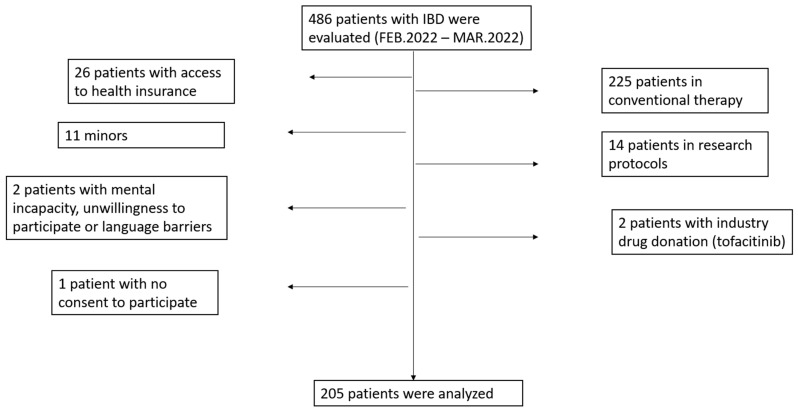
Flow chart of patients with IBD included in this study.

During the study period, 486 patients with IBD were evaluated at HCFMRP-USP. Of these, 205 patients met the inclusion criteria and answered the questionnaire during outpatient care. The other 281 patients were excluded from this study due to the following: patients with mild to moderate UC or CD using conventional therapy (immunosuppressants or 5-ASA derivatives or steroids) (*n* = 225); patients with access to biologics through health insurance, despite being followed-up in the public health system (*n* = 26); patients who received biologic drugs in research protocols (*n* = 14 (risankizumab, *n* = 4; upadacitinib, *n* = 3; ustekinumab, *n* = 7)); nonadult patients (<18 yo, *n* = 11); patients using tofacitinib due to drug donation by the pharmaceutical industry (*n* = 2); patients with mental incapacity, unwillingness to participate, or language barriers precluding adequate understanding or cooperation with the interviewer (*n* = 2); and patients that did not consent to participate in this study (*n* = 1). The flow chart is presented in Figure 1.

Most patients had CD (*n* = 161, 78.5%), with equal distribution between sexes (*n* = 104, 50.7%, female), and the mean age was 40.7 years (range, 19–75 years), with a mean disease duration of 11.9 years ( (2–24); 12.5 years (2–24) in CD and 9.7 years (2–20) in UC). Most patients with CD had a history of rectal stenosis or perianal fistulas (*n* = 76, 47.2%), and 96 patients (59.6%) with CD had a history of bowel resection. Eighty-seven patients (42.4%) were unemployed, and of these, forty patients (19.5%) had government assistance as a source of income. Regarding the medications in use, infliximab (*n* = 128, 62.5% (*n* = 101, CD; *n* = 27, UC)) was the most common medication in use, followed by adalimumab (*n* = 39, 19.0% (*n* = 32, CD; *n* = 7, UC)). Eight patients (3.9% (*n* = 8, CD)) were using certolizumab pegol, fourteen patients (6.8% (*n* = 3, CD; *n* = 11, UC)) were using vedolizumab, fifteen patients (7.3% (*n* = 15, CD)) were using ustekinumab, and one patient (0.5% (*n* = 1, UC)), with concomitant psoriatic arthritis, was using golimumab. These data are summarized in Table 1.

### 3.2. Patient Perceptions

Of all patients who completed the questionnaire, 172 (83.9%) reported at least one failed delivery of a biological in the last year. Among the patients who reported a lack of biologic delivery, forty-two patients (24.4%) reported a single shortage, forty-seven patients reported at least two shortages (27.3%), and seventy-eight patients reported that biologic delivery failed more than three times or that the drug had not yet been regularized (45.3%). The average time to regularize the distribution of biological medicine was up to 1 month in 44 cases (25.6%) and up to 2 months in 64 cases (37.2%). In 56 patients, there was a delay of 3 months or more to regularize the distribution of biological medicines, or there was still no normalization (32.6%). One hundred forty patients (68.3%) reported that pharmacists or high-cost pharmacy attendants gave explanations about the care with biological drugs and answered questions about the infusion and storage method. However, 53 (25.9%) patients reported that they did not receive this type of information from pharmacists or high-cost pharmacy attendants. Among the patients who reported a lack of biological drug dispensing (*n* = 172), 48 patients (27.9%) reported that pharmacists or high-cost pharmacy attendants did not clarify the reason or information about the lack of biological drug, while 113 patients (65.7%) reported that pharmacists or high-cost pharmacy attendants gave explanations on the subject. One hundred thirty-two patients (64.4%) tried to contact a high-cost pharmacy via phone, e-mail, or message during the period of non-dispensing of biologicals; however, sixteen of them (12.1%) were unable to make any contact. Contact with a high-cost pharmacy was easy in 68 cases (51.5%), but the contact was difficult in 48 cases (36.4%). More than 90% of patients (*n* = 179, 87.3%) thought that obtaining biological drugs was a bureaucratic process. These data are summarized in Table 2.

Regarding the delivery of the biological drug, 92 patients (44.9%) reported that they prefer it to continue as it is; that is, they prefer that the biological drugs be delivered by high-cost pharmacies. However, most patients (*n* = 105, 51.2%) preferred that biologics be delivered to the infusion clinic (*n* = 42, 40.0%) or to the hospital where they are treated (*n* = 27, 25.7%) or in either of the two (hospital or infusion clinic (*n* = 36, 34.3%). Only five patients (2.4%) reported that it is difficult to schedule the infusion of the biologic at the infusion clinic, with 183 patients (89.3%) reporting that it is easy. Among the 172 patients who reported a delay in drug delivery, 101 of them (58.7%) felt that the delay in providing biological drugs may have caused some type of damage in their treatment (for example, emergence or worsening of symptoms, loss of response, relapse, treatment failure, need to change the biologic, and emergence of adverse events). Finally, almost half of the patients (*n* = 101, 49.3%) were unaware of the pharmaceutical industry program for the biological drug in question. In addition, 151 patients (73.7%) thought that it was important that the laboratory should initiate treatment until the public health system regularizes biologic drug delivery or starts the patient’s treatment. These data are summarized in Table 3.

## 4. Discussion

To our knowledge, this is the first study of Brazilian patients with IBD that evaluated data regarding patient perception of access to biologicals using the SUS. Almost 84% of patients reported at least one failure in high-cost drug dispensation in the health care public system in the last year. In addition, almost 60% of them felt that the delay in providing biological drugs may have caused some type of impairment in their treatment. Our study showed that most patients accessing biologics through the SUS had trouble obtaining their biologic therapy at least once. CD and UC are IBDs with a relapsing–remitting presentation, and symptomatic exacerbations that have detrimental impacts on patient quality of life and are associated with a high-cost burden, especially in patients with the moderate to severe disease [3]. Those are patients with a higher severity of IBD; they cannot remain without any treatment, and the delay can be harmful to them. Thus, the time interval is essential in the treatment of IBD, especially when the patient is without any current treatment or will begin their first drug treatment for the disease [14].

In our study, patients had a high rate of perianal disease and a history of bowel resection (in CD), and most patients had left colitis or extensive colitis (in UC), with a long-standing disease duration in both diseases, confirming the clinical profile as moderate to severe in the patients with IBD included in this study. In CD, earlier treatment with biologics has been suggested as the best treatment approach to avoid disease progression and to reduce the risk of complications of the disease and disability.

Moreover, there is greater efficacy of biological therapy when this treatment is instituted early in the course of CD, known as the window of opportunity [14]. CD occurs early in the disease course, especially in the 1–2 years after diagnosis, before the patient develops structural damage [16]. In UC, the therapeutic approach should be prompt in the failure of conventional treatment and in the presence of poor prognostic factors, such as extensive colitis, younger age, and severe colitis [17]. Follow-up data from the CALM trial showed that inducing early profound remission in moderate to severe CD leads to a decreased risk of disease progression over a median time of 3 years, regardless of tight control or conventional management strategy [18]. A recent study showed that patients with IBD treated with biologics were significantly less likely to undergo bowel resection than those who never received biologics [19]. Thus, the delay in starting biological therapy in this profile of patients may reduce the effectiveness of the treatment and increase the risk of complications, such as increased rates of absence from work, worsening of perianal disease, and increased rates of hospitalizations and surgeries.

As a chronic disease, both CD and UC are associated with a cost of treatment [20]. However, not providing treatment leads to serious consequences, such as risk of disease activity, hospitalizations, surgeries, and higher cost of treatment. In general, hospitalizations are responsible for the large amount of the total direct costs falling on the health care system. The Crohn’s & Colitis Foundation’s Cost of IBD showed that the costs of care for IBD have increased in the last 5 years, and patients with IBD are increasingly incurring higher costs associated with health care utilization and workplace productivity losses [21]. Almost six out of seven patients with IBD reported at least one failure in biological distribution during the last year, and almost half of them reported at least three failures in the distribution of medicines in the period. The failure to supply biological drugs during maintenance within the correct period and, consequently, nonadherence to periodic regimens greatly increases the risk of immunogenicity and loss of response and infusion reactions to anti-TNF drugs [22]. When the biological therapy is interrupted, especially at the induction or when the drug level has dropped and is too low, restarting the therapy carries a high risk of developing antidrug antibodies and, therefore, losing response to the therapy. Thus, there is a higher risk of secondary loss of response and a need for a second or third anti-TNF drug [23,24]. Several studies have demonstrated a high risk of relapse after stopping biological drugs in IBD, which varies between 20 and 50% at 1 year and between 50 and 80% beyond 5 years. Only a minority of patients may not relapse over the mid-term, and these numbers clearly highlight that we should not stop therapy [25,26].

Despite Brazil having reached a record number of health insurance beneficiaries in 2021 with 48.4 million users, most of the Brazilian population does not have health insurance. Thus, in Brazil, patients without health insurance depend on the SUS to obtain conventional and biological therapies for IBD. The distribution of biological therapy is provided by the SUS through the CEAF. The Ministry of Health carries out centralized purchases and forwards them to the states that carry out the operational management. The patient makes the request by attaching the prescription, medical report, and exams related to the disease at CEAF pharmacies. This dossier is evaluated by state managers and, if it complies with the Clinical Protocols and Therapeutic Guidelines (PCDT) of the Federal Government, the supply of biologics and monitoring of treatment is authorized [27]. In CD, the PCDT does not offer any biologic other than anti-TNF drugs. Despite being approved in Brazil, new biologics such as ustekinumab and vedolizumab are not available for these patients [28,29]. Thus, there are no other options for treatment with biologics for patients with primary nonresponse to anti-TNF drugs or with contraindications for anti-TNF use, such as neoplasms, tuberculosis, and congestive heart failure. Patients with primary nonresponse to anti-TNF agents are less likely to respond to second-line non-TNF biologics [30].

For CD, the latest PCDT dates from 2017 and included the following biological drugs: infliximab, adalimumab, and certolizumab. Although vedolizumab has been registered by the National Health Surveillance Agency (ANVISA) since 2015 and ustekinumab since 2017, they are not part of this protocol for patients with CD. In addition, for UC, the latest PCDT dates from 2021 and included the following biological drugs: infliximab, vedolizumab, and, more recently (2022), tofacitinib. However, golimumab, adalimumab, and ustekinumab were not included. Thus, two biologics (vedolizumab and ustekinumab) used in the treatment of patients with CD and three biologics used in the treatment of UC (golimumab, adalimumab, and ustekinumab) are not included in the PCDT. Concomitantly, investment in public health care in Brazil has been decreasing annually [15]. Thus, government pharmacies have not been supplied as they should, including medicines used in the advanced care of patients with moderate to severe IBD.

The entire process, from prescription to infusion of the biological drug, could be simpler. Most patients in our study reported excessive bureaucracy to obtain biological medicines. First, there is a need for the patient to go to the pharmacy with the prescription and all the documentation. If any documents are missing, the patient must return to the hospital where the prescription was made and obtain the missing documents. It is worth mentioning that almost half of the patients in this study were unemployed or depended on government aid. For example, the delivery of documents online, directly between the prescribing doctor and the high-cost pharmacy, would speed up the process and minimize the risk of missing documents and incurring additional costs for the patients, thus facilitating the initiation of treatment. Regarding biological infusion, most patients agree that it is easier for the drug to be delivered directly to the hospital where it is followed-up or even to the infusion clinic. This would minimize the risk of spoiling the medicine, for example, due to errors in storage in the refrigerator.

Despite the importance of these data, our study had several limitations. First, despite patient perception being an important instrument in the assessment of the quality of care in health services, we did not assess any objective data, such as clinical flare, elevation in biomarkers, loss of response, or hospitalization rates. Second, despite being an IBD referral center with a high volume of patients, this was a single-center study with a short period of evaluation, and these data may not correspond to the situation in the rest of Brazil. However, due to the constant complaints of patients about the recurrent lack of distribution of biological medicines at the beginning of 2022, this study focused on a short period of evaluation so that society can be shown how the lack of dispensing of high-cost medicines can impact the course of IBD. The next step is to expand the research to more referral IBD centers, in a larger period of evaluation. Finally, this study focused on the evaluation of more severely ill patients, where the impact of lack of treatment may be greater; however, there is a need to expand the evaluation to patients with mild to moderate disease and with conventional therapy.

The journey of patients with IBD in Brazil has many obstacles [15]. First, the diagnosis of IBD is delayed due to disease-specific issues, and it is exacerbated by difficulty in accessing specialists who can perform an early diagnosis. Second, many patients also have difficulties accessing diagnostic tests, such as fecal calprotectin or colonoscopy. Third, patients have little or no access to a multidisciplinary health care team, even in IBD referral centers. Finally, the patients reported a lack of access to medicines, including biologics. In our study, most patients reported a lack of distribution during the last year and more than 3 months to regularize, and there were patients for whom the distribution did not normalize. Our study shows a worrisome scenario, where the lack of biological medicines, mainly due to problems in the logistics of the Ministry of Health and the State Health Departments, increases the risk of loss of response and infusion reactions to biological medicines, in addition to disease complications and disability in patients with IBD.

In summary, Brazilian IBD patients reported high rates of failure to dispense biological drugs by the national healthcare system within the previous one year. Our data highlight the need for improvement in this system for the correct supply of medication to avoid treatment failure and disease relapse.

## Figures and Tables

**Table 1:** Baseline clinical and demographic characteristics of 205 patients.

Characteristics	Results
Mean age, years (range)	40.7 (19–75)
**Gender, *n* (%)**	
Female	104 (50.7)
Male	101 (49.3)
**Occupation**	
Employed	112 (54.6)
Unemployed	87 (42.4)
Government assistance	40 (19.5)
Did not answer	6 (2.9)
**Crohn’s disease**	161 (78.5)
**Montreal Classification, *n* (%)**	
**Age at diagnosis**	
A1 (<17 years)	10 (6.2)
A2 (17–40 years)	101 (62.7)
A3 (>40 years)	50 (31.1)
**Disease location**	
L1 (ileal)	39 (24.2)
L2 (colonic)	35 (21.8)
L3 (ileocolonic)	82 (50.9)
L4 (upper gastrointestinal tract)	5 (3.1)
**Behavior**	
B1 (inflammatory)	53 (32.9)
B2/B3 (stenosing/penetrating)	108 (67.1)
**Ulcerative colitis**	44 (21.5)
Montreal Classification, *n* (%)	
E1	6 (13.6)
E2	12 (27.3)
E3	26 (59.1)
Mean disease duration, years (range)	11.9 (2–24)
CD	12.5 (2–24)
UC	9.7 (2–20)
History of perianal disease *	76 (47.2)
Previous bowel resection in CD	96 (59.6)
**Biologics in use, *n* (%)**	
Infliximab	128 (62.5)
Adalimumab	39 (19.0)
Certolizumab pegol	8 (3.9)
Golimumab	1 (0.5)
Vedolizumab	14 (6.8)
Ustekinumab	15 (7.3)

CD: Crohn’s disease; UC: ulcerative colitis. * Active or unactive perianal fistulas and/or rectal stenosis in patients with Crohn’s disease.

**Table 2:** Lack of biologics and patient perceptions.

Variable in Study	*n* (%)
**At least a lack of dispensation of biologics**	172 (83.9)
Single shortage	42 (24.4)
Two shortages	47 (27.3)
Three or more shortages	78 (45.3)
Did not answer	5 (2.9)
**Average time to regularize biologic distribution**	
One month	44 (25.6)
Two months	64 (37.2)
Three or more months	56 (32.6)
Did not answer	7 (2.4)
Infusion or storage explanations *	
Yes	140 (68.3)
No	53 (25.9)
**Explanations regarding the lack of dispensation**	
Yes	113 (65.7)
No	48 (27.9)
Did not answer	12 (5.8)
**Success in contacting the high-cost pharmacy**	
Yes	106 (80.3)
Easy contact	68 (51.5)
Difficult contact	48 (36.4)
No	16 (12.1)
Did not answer	6 (2.9)
**Bureaucratic process**	
Yes	188 (91.7)
No	12 (5.8)
Did not answer	5 (2.9)

* Pharmacists or high-cost pharmacy attendants explained about care with biological drugs and answered questions about the infusion and storage method.

**Table 3:** Patient perception of biologic prescription.

Variable	*n* (%)
**Delivery preference**	
High-cost pharmacy	92 (44.9)
Another place	105 (51.2)
Infusion clinic	42 (40.0)
Hospital	27 (25.7)
Infusion clinic or hospital	36 (34.3)
Did not answer	8 (3.9)
**Schedule the biologic at the infusion clinic**	
Easy	183 (89.3)
Difficult	5 (2.4)
Did not answer	17 (8.3)
Feeling of impairment of treatment *	
Yes	101 (58.7)
No	54 (31.4)
Did not answer	17 (9.9)
**Aware of the pharmaceutical industry program**	
Yes	95 (46.3)
No	101 (49.3)
Did not answer	9 (4.4)
**Importance of starting the treatment by industry**	
Yes	151 (73.7)
No	26 (12.7)
Did not answer	28 (13.6)

* Worsening of symptoms, loss of response, relapse, treatment failure, need to change the biologic, and emergence of adverse events.

## Data Availability

The datasets used and/or analyzed during the current study are available from the corresponding author on reasonable request.
